# Disability Prevalence and the Awareness-Utilization Gap for Disability Services in an Urban Community of Delhi, India: A Cross-Sectional Study

**DOI:** 10.7759/cureus.114574

**Published:** 2026-08-15

**Authors:** Swapnil T Sangle, Anand Vijay, Zil Gala, Dinesh Asokan, Sivasubramaniam SA

**Affiliations:** 1 Community Medicine, Government Medical College, Nashik, IND; 2 Community Medicine, Grant Government Medical College and Sir JJ Group of Hospitals, Mumbai, IND; 3 Community Medicine, Government Medical College and Hospitals, Mumbai, IND

**Keywords:** attitudes, cross-sectional studies, disability, health knowledge, health services accessibility, india, practice

## Abstract

Introduction: Disability-related welfare schemes form a critical component of India’s social protection architecture, yet community-level evidence on the gap between awareness and utilization in urban settings remains limited. This study aimed to estimate the prevalence of disability and its socio-demographic correlates and to assess awareness and utilization of disability-related services among adults in an urban community of Delhi, generating baseline data on the awareness-utilization gap for this setting.

Method: A community-based cross-sectional study was conducted in Palam, an urban resettlement colony in Delhi, from April 2020 to March 2021. Adults aged ≥25 years residing in the area for at least six months were included, and 1,246 individuals were selected by systematic random sampling. Disability - encompassing locomotor, visual, hearing, and speech impairments - was assessed using the Washington Group Short Set of Questions on Disability alongside self-report. Awareness and utilization of services across healthcare, transport, rehabilitation, and social welfare domains were captured through face-to-face interviews using a pretested semi-structured schedule. Data were analyzed using descriptive statistics and chi-square or Fisher’s exact tests.

Results: Disability was identified in 87 of 1,246 participants (6.9%; 95% CI: 5.6%-8.4%). Prevalence was significantly higher among males (59/655; 9.0%) than females (28/591; 4.7%; p = 0.003) and varied significantly by socio-economic status (p = 0.001). Locomotor disability was most common (47/87; 54.0%), followed by hearing (18/87; 20.7%), speech (18/87; 20.7%), and visual disabilities (17/87; 19.5%); some participants reported multiple impairment types, and percentages accordingly sum to more than 100%. Awareness was universal for disability certification, pensions, and transport fare concessions (100% each), with somewhat lower awareness of seat reservation (78/87; 89.7%) but very low for the Unique Disability ID (UDID) card (6.9%), health insurance (6.9%), and loan benefits (6.9%). Utilization was highest for seat reservation (73.6%), disability certification (67.8%), and pensions (55.2%), while no participant reported utilizing health insurance or loan benefits. As data collection coincided with the COVID-19 pandemic (April 2020-March 2021), reported utilization rates likely reflect pandemic-related service disruptions and should not be taken as indicative of baseline access.

Conclusion: Disability prevalence substantially exceeded national census estimates. Although awareness of several entitlements was high, utilization was consistently lower, with the widest gaps for health insurance, loan benefits, and vocational training. Procedural complexity and implementation failures, rather than information deficits, appear to be the primary barriers to service access. Pandemic-era disruptions likely contributed to the magnitude of the observed gaps. Bridging this divide requires simplified access pathways, targeted IEC (Information, Education, and Communication) campaigns for newer schemes such as the UDID card, and integration of disability services into routine primary healthcare.

## Introduction

Disability is a significant global public health and social justice concern, affecting more than one billion people worldwide - approximately 16% of the global population [1]. Persons with disabilities face disproportionate barriers to healthcare, education, employment, and social participation, adversely affecting quality of life and impeding progress toward universal health coverage, particularly in low- and middle-income countries where health systems often remain fragmented and insufficiently responsive [1]. For the purposes of this study, disability is operationally defined to encompass locomotor, visual, hearing, and speech impairments, consistent with the functional limitation framework of the Washington Group Short Set of Questions on Disability described in the Materials and Methods section.

In India, the 2011 Census reported a disability prevalence of 2.2%; however, community-based studies have reported substantially higher estimates, often in the range of 5% to 10%, suggesting that administrative data substantially underestimate the true burden due to stigma, restrictive definitional criteria, and underreporting [2]. Ongoing demographic aging is expected to further increase disability prevalence, placing additional demands on health and social systems [2]. In response, India has enacted several legislative and programmatic measures - including the Rights of Persons With Disabilities Act 2016 (RPWD Act 2016), the Unique Disability ID project, and the Accessible India Campaign - aimed at improving inclusion and access to disability-related services [3]. Despite these initiatives, legislative provisions do not consistently translate into effective utilization at the community level.

A growing body of literature describes an “awareness-utilization gap,” wherein persons with disabilities may know about entitlements such as disability certification, pensions, or transport concessions yet remain unable to use them because of procedural complexity, administrative delays, stigma, or inadequate infrastructure [4]. Studies from Indian settings have reported utilization rates substantially lower than awareness across multiple service domains [4]. Utilization of rehabilitation services and assistive technologies remains particularly low despite documented need, reflecting persistent systemic inequities in service delivery [5].

Community-based evidence from India examining disability prevalence alongside awareness and utilization of services across multiple domains simultaneously remains limited. Much of the existing literature is hospital-based or restricted to specific impairment groups, offering limited insight into how disability entitlements function in real-world community settings. Urban resettlement colonies present a particularly relevant context for such enquiry: residents live in proximity to health facilities and administrative offices yet frequently face barriers related to documentation requirements, procedural complexity, and economic constraints. The present study was therefore conducted to estimate the prevalence of disability and its socio-demographic correlates and to assess awareness and utilization of disability-related services in such a setting, with the aim of identifying specific service domains where implementation gaps are most severe. By quantifying the magnitude of the awareness-utilization gap across service domains during this period, the study seeks to generate baseline data that can serve as a reference point for monitoring the translation of disability policy into community-level access over time.

## Materials and methods

Study design and setting

A community-based cross-sectional study was conducted from April 2020 to March 2021 in Palam, Delhi, an urban resettlement area characterized by high population density and a mixed socio-economic profile. One of five catchment areas of Palam was selected using simple random sampling by the lottery method, and a Scheduled Caste resettlement colony (Harijan Basti) was identified as the study area. As per the annual primary health center survey for 2020-2021, this area had a total population of 11,047 residing in 1,700 households.

The study was conducted during the COVID-19 pandemic, a period characterized by successive lockdown phases, intermittent restrictions on movement, and severe disruptions to non-essential health and social services in Delhi. Data collection adhered to COVID-19-appropriate safety protocols throughout, including mandatory masking, physical distancing, and use of hand sanitizers during all household visits. Where direct household entry was not feasible during active restriction periods, interviews were conducted at the doorway or in an open outdoor area on the premises. Fieldwork was temporarily suspended during strict lockdowns and resumed when government guidelines permitted household visits. The potential impact of pandemic-era service disruptions on the utilization rates reported in this study is discussed in the Limitations section.

Study population and eligibility

The study population comprised adults aged ≥25 years who had been residing in the study area for at least six months. The age criterion of ≥25 years was chosen to capture adults who have typically completed formal education and entered the workforce or independent living, and who are therefore the primary intended beneficiaries of disability entitlements related to employment, financial assistance, and social welfare. The sampling unit was the household, and all eligible family members aged ≥25 years within selected households were included as study units. Individuals unavailable despite at least three household visits, and those who declined to provide informed consent, were excluded.

Sample size and sampling technique

Sample size was calculated using the formula,

\( n = \frac{Z^2pq}{d^2} \)

where Z = 1.96 (95% confidence level), p = assumed non-utilization rate of 55% based on estimates from comparable studies in the Indian disability literature [4], q = 0.45, and d = absolute error of 11%. This yielded a minimum of 87 persons with disability. Assuming a disability prevalence of approximately 7% based on prior community-based estimates in similar urban settings [2], the total sample required to yield 87 persons with disability was 1,243 individuals. Accounting for an anticipated 10% non-response rate, the target sample was 1,367; 1,246 individuals were ultimately enrolled, as the actual non-response rate was lower than expected. Participants were selected from an updated household list by systematic random sampling at a fixed interval derived from the total household count.

Data collection and study tools

Data were collected through face-to-face interviews using a self-designed, pretested, semi-structured interview schedule. Where individuals were unable to respond, interviews were conducted with their primary caregiver. The schedule captured socio-demographic characteristics, disability type and certification status, awareness of disability-related services, and utilization of these services across healthcare, transport, rehabilitation, and social welfare domains. Socio-economic status was assessed using the modified Kuppuswamy scale [6] with the Consumer Price Index for 2020 [7].

The interview schedule was developed through a systematic process: (1) a preliminary version was drafted based on a review of the existing literature on disability services in India and consultation with community medicine and disability rehabilitation specialists at the institution; (2) content validity was established through expert review by three senior faculty members, each with more than ten years of research and teaching experience in community medicine, epidemiology, and disability research, respectively, with the schedule refined based on their feedback; (3) the schedule was translated from English into Hindi and back-translated by independent translators to ensure linguistic equivalence and cultural appropriateness; (4) pretesting was conducted with 20 adults from a neighbouring community not included in the study area, resulting in simplification of questions, refinement of response options, and reduction of interview duration from approximately 30 to 20 minutes; and (5) face validity and comprehensibility were assessed during pretesting through direct participant feedback on clarity and relevance. The complete interview schedule is provided in the Appendices. The schedule was derived primarily through a deductive approach: item content was anchored to pre-existing, validated frameworks and instruments - the Washington Group Short Set of Questions on Disability [8] for disability screening, the modified Kuppuswamy scale for socio-economic classification, and the service categories recognized under the RPWD Act 2016 for the awareness and utilization items - rather than emerging inductively from open-ended community input. An inductive element was nonetheless incorporated at the margins: wording, response options, and question sequencing were iteratively refined based on participant feedback during pretesting (step 4 above), and the open-ended barrier questions in Section E were designed to allow inductive identification of barrier themes not anticipated by the structured framework.

All interviews were conducted by the principal investigator, a faculty member in community medicine. Prior to data collection, repeated mock interview sessions were conducted with colleagues and supervisors to refine the interview schedule, standardize probing techniques, and assess question clarity. Pilot interviews were also carried out with participants from the neighbouring community during the pretesting phase; responses were used to finalize wording and interview flow before commencement of the main study. Confidentiality was assured before sensitive questions, and neutral, non-leading probes were used throughout to minimise social desirability bias. “Don’t know” responses for awareness questions were coded separately and treated as “not aware” in the analysis; missing item data were handled by listwise deletion.

Screening for disability was performed using the Washington Group Short Set of Questions on Disability [8] to identify functional limitations. Detailed information was also collected on challenges faced in accessing services.

Clinical examination

All individuals identified with disability underwent a clinical examination to verify and characterize disability type. This included anthropometric assessment, general physical and system-wise examination, visual acuity assessment using a Snellen chart, colour vision testing using the Ishihara chart, and hearing assessment using tuning fork tests (a screening rather than a formal diagnostic measure such as audiometry). Blood pressure was measured using an aneroid sphygmomanometer following standard procedures. The examination was conducted to characterize the clinical profile of participants with disability and to verify self-reported disability type; detailed clinical findings beyond disability characterization are beyond the scope of this paper and will be reported separately.

Definitions

Disability was defined as a self-reported long-term impairment affecting locomotion, vision, hearing, or speech that resulted in functional limitation. This operational definition encompasses locomotor, visual, hearing, and speech impairments, consistent with the functional limitation framework of the Washington Group Short Set of Questions on Disability [8], which assesses these domains jointly. This functional approach was adopted in preference to the categorical, certification-based definitions specified under the RPWD Act 2016 for two reasons. First, a central objective of this study was to estimate community-level disability burden independent of formal certification status; anchoring ascertainment to RPWD Act categories would have restricted identification to individuals already engaged with the certification system, understating the true burden in a manner similar to the census-based undercounting this study sought to address. Second, using certification-based criteria to define the study population would have introduced circularity when subsequently examining awareness and utilization of the certification process itself as a primary outcome. The RPWD Act 2016 categories and associated entitlements nonetheless directly informed the content of the awareness and utilization items in Section C and D of the interview schedule (see Appendices). Awareness was defined as knowledge of the existence of a specific disability-related service; utilization was defined as actual use of that service by the respondent at any point.

Study variables and outcome measures

The primary outcome measures were awareness of disability-related services and utilization of these services among individuals with disability. Secondary outcomes included the prevalence of disability among adults aged ≥25 years and associations of socio-demographic factors with disability and utilization. Explanatory variables included age, sex, education, occupation, socio-economic status, caste, religion, and disability type.

Statistical analysis

Data were entered into Microsoft Excel (Microsoft Corporation, Redmond, CA, USA) and analysed using IBM SPSS Statistics, version 21 (IBM Corp., Armonk, NY) - the version available at the institution at the time of data collection. Descriptive statistics summarized characteristics and outcomes as N (%). Associations between categorical variables were assessed using the chi-square test; Fisher’s exact test was applied where expected cell counts were below five. A p-value < 0.05 was considered statistically significant. No correction for multiple comparisons was applied, consistent with the exploratory and descriptive nature of the study. Multivariable logistic regression to identify independent predictors of utilization was planned but was not feasible due to very small cell sizes for several outcomes (e.g., only two participants utilized educational reservation; only one held a UDID card). Results should be interpreted as descriptive bivariate associations, and potential confounding by correlated socio-demographic variables should be borne in mind.

Ethical considerations

The study was approved by the Institutional Ethics Committee, Lady Hardinge Medical College & Associated Hospitals, New Delhi (Approval No. LHMC/IEC/Thesis/2019/3, dated October 28, 2019). Written informed consent was obtained from all participants before data collection. For participants unable to provide written consent due to cognitive or communication impairments, consent was obtained from a legally authorized representative (primary caregiver), and assent was elicited from the participant where feasible. The study was anonymized, and confidentiality and privacy were maintained throughout.

## Results

Of 1,367 individuals identified through systematic random sampling from the household list, 1,246 (91.2%) completed the interview. Non-participation (n = 121; 8.8%) was attributed to individuals being unavailable despite three household visits or refusal to provide informed consent; this was marginally lower than the 10% non-response rate anticipated during sample size calculation.

The socio-demographic profile of participants is presented in Table 1. A total of 1,246 adults aged 25 years and above were enrolled, with a slight male predominance (52.6%). The mean age was 40.7 ± 13.4 years, and the largest proportion belonged to the 25-34 year age group (40.7%). Most participants were married (78.3%). Nearly one-quarter had no formal education or only primary schooling, while 22.4% were graduates or postgraduates. The majority were Hindu (85.3%), belonged to the Scheduled Caste social category (45.3%), and were from upper-lower or lower-middle socio-economic strata, reflecting a predominantly lower socio-economic urban population.

**Table 1:** Socio-demographic characteristics of study participants (N = 1,246) *Others include Sikh, Buddhist, Jain, and other religious minorities. Educational categories follow the schema used in the study interview schedule (“Less than middle school” = Classes 1-4; “Middle school” = Classes 5-7). OBC = Other Backward Classes; SC = Scheduled Castes; ST = Scheduled Tribes

Variable	Category	n (%)
Sex	Male	655 (52.6)
	Female	591 (47.4)
Age group (years)	25-34	508 (40.7)
	35-44	291 (23.3)
	45-54	227 (18.2)
	55-64	126 (10.2)
	≥65	94 (7.6)
Marital status	Unmarried	211 (16.9)
	Married	975 (78.3)
	Widow/Widower	53 (4.2)
	Divorced	7 (0.6)
Education status	Illiterate	220 (17.7)
	Less than middle school	103 (8.3)
	Middle school	205 (16.5)
	High school	266 (21.3)
	Intermediate/Diploma	172 (13.8)
	Graduate	200 (16.0)
	Postgraduate	80 (6.4)
Religion	Hindu	1063 (85.3)
	Muslim	129 (10.4)
	Christian	30 (2.4)
	Others*	24 (1.9)
Socio-economic status	Lower	19 (1.5)
	Upper-lower	556 (44.6)
	Lower-middle	502 (40.3)
	Upper-middle	156 (12.5)
	Upper	13 (1.1)
Social category	General	337 (27.0)
	OBC	321 (25.8)
	SC	565 (45.3)
	ST	23 (1.8)

The overall prevalence of disability was 6.9% (87/1,246; 95% CI: 5.6%-8.4%), as shown in Table 2. Prevalence was significantly higher among males (59/655; 9.0%; 95% CI: 6.8%-11.2%) than females (28/591; 4.7%; 95% CI: 3.0%-6.5%; χ² = 8.72, p = 0.003). This gender difference may reflect greater male exposure to occupational hazards and injury risk, alongside potential underreporting among women attributable to stigma or reduced access to diagnostic services, though these explanations remain inferential in the absence of direct data [2].

**Table 2:** Prevalence of disability by gender among study participants (N = 1,246) Percentages are column percentages within each gender category. Association tested using Pearson’s chi-square test (χ² = 8.72, df = 1, p = 0.003). Prevalence: males 9.0% (95% CI: 6.8%–11.2%); females 4.7% (95% CI: 3.0%–6.5%); overall 6.9% (95% CI: 5.6%–8.4%).

Gender	With disability (n)	With disability (%)	Without disability (n)	Without disability (%)
Male	59	9.0	596	91.0
Female	28	4.7	563	95.3
Total	87	6.9	1159	93.1

Age-specific disability prevalence is presented in Table 3. The highest prevalence was observed in the 55-64 year age group (11.9%), and the lowest in the 45-54 year group (3.9%). The overall association was not statistically significant (p = 0.09). The notably low prevalence in the 45-54 year group relative to adjacent younger and older groups is biologically unexpected and may reflect survival bias (individuals with severe early-onset disability may have had excess mortality before reaching this age), selective out-migration from the study area, or sampling variability given the small number of affected individuals in that group. This finding should be interpreted with caution. Because age-specific prevalence was non-monotonic across the age groups, a formal test for linear trend was not appropriate.

**Table 3:** Prevalence of disability by age group (N = 1,246) χ² = 8.01, df = 4, p = 0.09. The lower prevalence observed in the 45-54-year age group relative to adjacent groups is discussed in the text.

Age group (years)	With disability n (%)	Without disability n (%)	Total n (%)
25-34	37 (7.3)	471 (92.7)	508 (40.7)
35-44	20 (6.9)	271 (93.1)	291 (23.3)
45-54	9 (3.9)	218 (96.1)	227 (18.2)
55-64	15 (11.9)	111 (88.1)	126 (10.2)
≥65	6 (6.4)	88 (93.6)	94 (7.6)
Total	87 (6.9)	1159 (93.1)	1246 (100)

The distribution of disability by socio-economic status is shown in Table 4. Prevalence was highest among participants from the lower class (15.8%) and lowest in the lower-middle class (3.8%), with a statistically significant overall association (p = 0.001). Notably, the combined upper-middle and upper SES group showed a prevalence of 11.2%, higher than expected relative to the middle strata. This U-shaped pattern may reflect an older age distribution among higher SES participants (associated with greater longevity and consequently more age-related disability), better ascertainment through healthcare access, or sampling variability given the small number of participants in the upper SES category (n = 13).

**Table 4:** Prevalence of disability by socio-economic status (N = 1,246) χ² = 16.32, df = 3, p = 0.001. †Upper-middle and upper categories combined due to small sample size in the upper category (n = 13, 1.1%).

Socio-economic status	With disability n (%)	Without disability n (%)	Total n (%)
Lower	3 (15.8)	16 (84.2)	19 (1.5)
Upper-lower	46 (8.3)	510 (91.7)	556 (44.6)
Lower-middle	19 (3.8)	483 (96.2)	502 (40.3)
Upper-middle and upper†	19 (11.2)	150 (88.8)	169 (13.6)
Total	87 (6.9)	1159 (93.1)	1246 (100)

The association between educational status and disability is presented in Table 5. Prevalence was highest among individuals who were illiterate or had primary education (9.6%) and lowest among those educated to the middle or intermediate level (5.6%). The association was not statistically significant (p = 0.07), though the p-value approached conventional thresholds and a larger sample might have revealed a significant gradient.

**Table 5:** Prevalence of disability by educational status (N = 1,246) χ² = 5.30, df = 2, p = 0.07. Educational status was collapsed post hoc from seven categories into three (illiterate/primary, middle-to-intermediate, and graduate/postgraduate) to ensure adequate cell counts for the association analysis.

Educational status	With disability n (%)	Without disability n (%)	Total n (%)
Illiterate and primary	31 (9.6)	292 (90.4)	323 (26.0)
Middle to intermediate	36 (5.6)	607 (94.4)	643 (51.6)
Graduate and postgraduate	20 (7.1)	260 (92.9)	280 (22.4)
Total	87 (6.9)	1159 (93.1)	1246 (100)

Disability type and service awareness and utilization are presented in Table 6. Locomotor disability was the most common type (47/87; 54.0%), followed by hearing (18/87; 20.7%), speech (18/87; 20.7%), and visual disabilities (17/87; 19.5%). Given that some participants reported more than one impairment type, these percentages sum to more than 100%. Clinical examination confirmed the disability type in all identified individuals; detailed clinical findings are beyond the scope of this paper.

**Table 6:** Awareness and utilization of disability-related services among persons with disability (n = 87) Gap (%) calculated as (Awareness − Utilization) ÷ Awareness × 100. For services with awareness <87/87, the gap percentage represents the proportion of aware individuals who did not utilise the service. UDID = Unique Disability ID

Service	Aware n (%)	Utilized n (%)	Gap (%)	Domain
Disability certificate	87 (100)	59 (67.8)	32.2	Healthcare
UDID card	6 (6.9)	1 (1.1)	84.0	Healthcare
Health insurance	6 (6.9)	0 (0.0)	100.0	Healthcare
Transport concession	87 (100)	48 (55.2)	44.8	Transport
Seat reservation	78 (89.7)	64 (73.6)	17.9	Transport
Disability-friendly transport	58 (66.7)	20 (22.9)	65.7	Transport
Assistive devices/aids	45 (51.7)	32 (36.8)	28.8	Rehabilitation
Vocational training	18 (20.7)	7 (8.0)	61.3	Rehabilitation
Disability pension	87 (100)	48 (55.2)	44.8	Social welfare
Reservation in education	23 (26.4)	2 (2.3)	91.3	Social welfare
Loan benefits	6 (6.9)	0 (0.0)	100.0	Social welfare

Awareness was consistently higher than utilization across all service domains. Universal awareness was observed for disability certification, transport fare concessions, and pensions (100% each). Awareness of seat reservation, while high (78/87; 89.7%), was somewhat lower than for fare concessions. Awareness of newer or more administratively complex schemes was markedly low: only six participants (6.9% each) had heard of the UDID card, health insurance for persons with disabilities, or loan benefits. Utilization was highest for seat reservation in public transport (64/87; 73.6%; 95% CI: 64.3%-82.9%), disability certification (59/87; 67.8%; 95% CI: 58.0%-77.6%), and pensions (48/87; 55.2%; 95% CI: 44.8%-65.6%). No participant reported utilizing health insurance or loan benefits. The widest awareness-utilization gaps were observed for health insurance and loan benefits (100% each), educational reservations (91.3%), and vocational training (61.3%). Due to small cell sizes across utilization outcomes, multivariable logistic regression was not feasible; bivariate associations with socio-demographic variables are presented in Tables 2-5, and potential confounding should be considered when interpreting these findings. Awareness and utilization are reported for the aggregate group of persons with disability; the small number of individuals within each impairment category precluded a reliable subgroup analysis of awareness and utilization by disability type.

## Discussion

This community-based study provides empirical evidence on the burden of disability and the functioning of disability-related service delivery in an urban resettlement setting of Delhi.

Prevalence of disability in community context

The observed disability prevalence of 6.9% substantially exceeded national census estimates, reinforcing long-standing concerns that administrative data underestimate the true disability burden in community settings [9]. This discrepancy is consistent with findings from other Indian community-based studies reporting prevalence in the range of 5% to 10% [2, 10], and most likely reflects the methodological advantage of functional screening using the Washington Group Short Set over the narrow legal and administrative definitions relied upon in national enumeration. Community-level screening identifies individuals experiencing daily activity limitations even in the absence of formal certification and therefore yields more realistic estimates of disability burden in urban populations [11].

Sociodemographic correlates of disability

The higher prevalence among males aligns with patterns reported in other Indian settings and may reflect greater male exposure to occupational injuries and hazardous working environments, as well as potential underreporting among women due to stigma or reduced access to diagnostic services [2]. These explanations remain inferential, however, as the study did not directly measure occupational exposures, reporting behavior, or healthcare utilization.

A significant association between lower socio-economic status and higher disability prevalence underscores the well-recognized bidirectional relationship between poverty and disability: individuals from economically disadvantaged backgrounds are more vulnerable to disabling conditions through hazardous living and working environments and delayed treatment, while disability simultaneously entrenches poverty by restricting employment opportunities and generating healthcare expenditure [12]. The socio-economic gradient observed in this study highlights disability as both a product and a driver of social disadvantage in urban informal settlements [13].

An unexpected finding was the elevated disability prevalence in the combined upper-middle and upper SES group (11.2%), approaching the rate in the lowest SES stratum. This U-shaped pattern may reflect an older age distribution among higher SES participants (with greater longevity and consequently more age-related disability), better diagnostic ascertainment through healthcare access, or sampling variability given the very small number of participants in the upper SES category. This finding warrants cautious interpretation.

The awareness-utilization gap

Despite universal or near-universal awareness of disability certification (100%), transport fare concessions (100%), and pensions (100%), utilization of these services ranged from only 55.2% to 67.8%. This persistent discordance between knowing about a service and actually using it suggests that awareness is necessary but not sufficient for uptake. Procedural complexity, documentation requirements, physical inaccessibility of administrative offices, and indirect costs such as time and travel are plausible contributors, consistent with Andersen’s behavioural model of health service use and other established frameworks that recognize enabling resources - including ease of access, transportation, and administrative facilitation - as critical determinants of utilization beyond awareness alone [4, 13]. The findings are consistent with barriers being primarily operational rather than informational, though this interpretation rests on inference, as direct data on barriers were not formally analyzed in this study.

Services with widest gaps: policy implications

The widest awareness-utilization gaps were for health insurance and loan benefits (100% each, meaning none of the few aware individuals utilized them), educational reservations (91.3%), and vocational training (61.3%). These services share a feature: they require multi-step administrative processes and coordination across sectors - health, finance, education, and social welfare - which disproportionately disadvantage persons with disabilities from lower socio-economic backgrounds [14]. Even where awareness exists, limited institutional facilitation and fragmented implementation appear to impede meaningful access [5].

Two specific findings merit particular attention from a policy standpoint. First, awareness of the Unique Disability ID card was strikingly low (6/87; 6.9%), with only one participant actually holding one. The UDID project, introduced under the RPWD Act 2016, was designed to unify disability certification and simplify access to entitlements across sectors. Its near-complete absence from awareness in this urban Delhi community - which is not geographically remote from administrative offices - points to a serious implementation gap in outreach and registration. Second, no participant reported utilizing health insurance for disability, and awareness was confined to six individuals (6.9%). National health protection schemes include provisions for persons with disabilities, yet these are clearly not reaching this population, suggesting either that targeted awareness campaigns have not reached this community or that enrollment processes are perceived as prohibitively complex.

Conversely, seat reservation in public transport and assistive devices had comparatively narrow gaps (17.9% and 28.8%, respectively). These services share an important structural characteristic: they can be accessed through relatively straightforward pathways without extensive documentation or multi-agency coordination. Seat reservation is an automatic entitlement on public transport, while assistive devices are often distributed directly through hospitals and government camps. This contrast suggests that streamlining and decentralizing access procedures - rather than introducing new schemes - may be the more effective lever for improving utilization.

Taken together, the findings suggest that India’s disability policy framework has achieved meaningful progress in creating awareness of traditional entitlements but remains constrained in translating policy into utilization, particularly for newer and more administratively complex services. Strengthening implementation efficiency, simplifying access procedures, and embedding disability service facilitation within routine primary healthcare - including through trained frontline health workers - are likely to be more impactful than launching additional schemes. Digital platforms and civil society partnerships could further support outreach and enrollment [5].

Limitations

This study has several limitations. The cross-sectional design precludes causal inference, and all reported associations are bivariate and may be confounded. Disability ascertainment relied on self-report supplemented by the Washington Group Short Set, which is subject to recall and social desirability bias; stigma may have led to underreporting, and awareness of socially valued services may be overstated. Disability certification was verified by sighting the official document where it was available at the time of interview, but a proportion of certification and service-utilization data relied on respondent self-report, which may introduce misclassification. The operational definition used here - encompassing locomotor, visual, hearing, and speech impairments - is broader than some administrative classifications and should be kept in mind when comparing findings across studies. Very small cell sizes for several outcomes precluded multivariable regression and yielded wide confidence intervals for rare utilization estimates. Most critically, data collection coincided with the COVID-19 pandemic (April 2020-March 2021), a period of successive lockdowns and severe disruptions to non-essential health and social services in Delhi; the utilization rates reported here almost certainly represent pandemic-period minima and should not be extrapolated as baseline access patterns. Finally, the single-site design (one urban resettlement colony in Delhi) limits generalizability to other urban settings, rural areas, or regions with different levels of disability welfare scheme implementation.

Recommendations

Several targeted measures are recommended to address the gaps identified. Awareness campaigns should prioritize newer and underutilized schemes - particularly the UDID card and health insurance for persons with disability - which showed near-zero awareness in this community despite being mandated under the RPWD Act 2016. Disability certification and UDID registration should be decentralized to the primary healthcare level to reduce the administrative burden on persons with disabilities. Vocational training and educational support services should be actively linked to primary healthcare delivery, with frontline workers (ASHAs, community health officers) trained as disability service navigators. Finally, a follow-up study under non-pandemic conditions is needed to establish baseline awareness and utilization rates and to assess the impact of policy changes implemented since 2021.

## Conclusions

This community-based study conducted in an urban resettlement colony of Delhi found a disability prevalence of 6.9%, substantially exceeding national census estimates, with higher prevalence among males and individuals from lower socio-economic groups. Although awareness of established entitlements, including disability certification, pensions, and transport concessions, was high, their actual utilization was consistently lower across service domains. The largest gaps between awareness and utilization were observed for health insurance, loan benefits, educational reservations, and the UDID card, indicating that awareness of available entitlements does not necessarily translate into their effective uptake at the community level, and that procedural and implementation barriers, rather than information deficits, are the primary constraint on access.

The finding that services with simpler, more direct access pathways, namely seat reservation in public transport and provision of assistive devices, had comparatively narrower gaps lends support to the conclusion that streamlining access procedures may be more effective than creating new schemes. As detailed previously, the COVID-19 pandemic context of data collection is a critical caveat for interpreting the absolute magnitude of these findings. Bridging the awareness-utilization gap will therefore require the multi-pronged approach discussed earlier, centred on simplifying access pathways and strengthening implementation rather than introducing new schemes, so that persons with disability can effectively access the entitlements guaranteed to them under law.

## Appendices

Semi-structured interview schedule

Study: Awareness and Utilization of Disability Services in Urban Delhi

Date of Interview: __________________     Interviewer ID: __________________

Household ID: __________________          Participant ID: __________________

Section A: Socio-demographic Information

1.        Name: _________________________________ (to be anonymized during data entry)

2.        Age (in completed years): _______

3.        Sex:  ☐ Male   ☐ Female   ☐ Other

4.        Marital status:  ☐ Unmarried   ☐ Married   ☐ Widow/Widower   ☐ Divorced   ☐ Separated

5.        Educational status:

           ☐ Illiterate   ☐ Less than middle school (Class 1-4)   ☐ Middle school (Class 5-7)

           ☐ High school (Class 8-10)   ☐ Intermediate/Diploma   ☐ Graduate   ☐ Postgraduate

6.        Occupation (circle appropriate category for Kuppuswamy scoring):

           1 - Professional (doctor, engineer, lawyer, senior government officer)

           2 - Semi-professional (teacher, nurse, junior officer)

           3 - Clerical / shop owner / farmer

           4 - Skilled worker

           5 - Semi-skilled worker

           6 - Unskilled worker / unemployed / homemaker

           Occupation of head of family (category): _______

7.        Religion:  ☐ Hindu   ☐ Muslim   ☐ Christian   ☐ Others (specify): ____________

8.        Caste/Social category:  ☐ General   ☐ OBC   ☐ SC   ☐ ST

9.        Household size (number of persons): _______

10.      Duration of residence in study area: _______ years

11.      Modified Kuppuswamy Socioeconomic Scale (2020):

           a) Education of head of family: [Score: ___]

           b) Occupation of head of family (from Q6): [Score: ___]

           c) Monthly family income from all sources (INR): _______ [Score: ___]

           Total score: _____

           SES Category:  ☐ Upper   ☐ Upper-middle   ☐ Lower-middle   ☐ Upper-lower   ☐ Lower

Section B: Disability Screening

12.      Washington Group Short Set of Questions on Disability [8]

           Instructions: Ask each question exactly as worded. Record response code: 1 = No difficulty, 2 = Some difficulty, 3 = A lot of difficulty, 4 = Cannot do at all.

           WGQ1. Do you have difficulty seeing, even if wearing glasses?                          [___]

           WGQ2. Do you have difficulty hearing, even if using a hearing aid?                    [___]

           WGQ3. Do you have difficulty walking or climbing steps?                              [___]

           WGQ4. Do you have difficulty remembering or concentrating?                           [___]

           WGQ5. Do you have difficulty with self-care, such as washing all over or dressing?   [___]

           WGQ6. Using your usual language, do you have difficulty communicating,

                  for example understanding or being understood by others?                      [___]

           ► SKIP PATTERN: If all WGQ responses = 1 (No difficulty) AND Q13 = No → proceed to end of interview

13.      Self-reported disability:

           Do you have any long-term disability (lasting >6 months) affecting your daily activities?

           ☐ Yes → Continue to Q14          ☐ No → Skip to end of interview

14.      If YES, type of disability (tick all that apply):

           ☐ Locomotor   ☐ Visual   ☐ Hearing   ☐ Speech   ☐ Other: _______________

           If multiple, which causes the most difficulty in daily life? _______________

15.      Do you have an official Disability Certificate?   ☐ Yes   ☐ No

           If Yes: % of disability: _____   Issuing authority: _____________   Date: _____________

           Certificate verified: ☐ Yes (sighted)   ☐ No (self-report only)

Section C: Awareness of Disability-related Services

Instructions: Ask “Have you heard of [service]?” for each item. If “Don’t know,” probe once with a brief neutral description. Response codes: 1 = Yes (aware), 2 = No (not aware), 9 = Don’t know (to be treated as “not aware” in analysis).

Healthcare domain

16.      Disability Certificate [___]

17.      Unique Disability ID (UDID) Card [___]

18.      Health Insurance for Persons with Disabilities (e.g., PM-JAY, state schemes) [___]

Transport domain

19.      Transport concession / reduced fare in buses and trains [___]

20.      Reserved seating in public transport [___]

21.      Disability-friendly transport facilities [___]

Rehabilitation domain

22.      Assistive devices / aids (wheelchair, crutches, hearing aid, etc.) [___]

23.      Vocational training programs for persons with disabilities [___]

Social welfare domain

24.      Disability pension [___]

25.      Reservation in education (reserved seats in schools and colleges) [___]

26.      Loan benefits / financial assistance schemes [___]

Section D: Utilization of Disability-related Services

Instructions: For each service the respondent is AWARE of (Section C = 1), ask: “Have you ever used or received this service?” Response codes: 1 = Yes, 2 = No, 0 = Not applicable (not aware - skip if Section C = 2 or 9).

27.      Disability Certificate [___]   Verified: ☐   Frequency: ☐ Regularly  ☐ Occasionally  ☐ Once only

28.      UDID Card [___]              Verified: ☐

29.      Health Insurance [___]

30.      Transport concession [___]   Verified: ☐   Frequency: ☐ Regularly  ☐ Occasionally  ☐ Once only

31.      Seat reservation [___]        Frequency: ☐ Regularly  ☐ Occasionally  ☐ Once only

32.      Disability-friendly transport [___]

33.      Assistive devices [___]       Type: __________   Currently functional: ☐ Yes  ☐ No  ☐ Partially

34.      Vocational training [___]

35.      Disability pension [___]      Verified: ☐   Frequency: ☐ Regularly  ☐ Occasionally  ☐ Once only

36.      Educational reservation [___]

37.      Loan benefits [___]

Section E: Barriers to Utilization

Instructions: For each service known but not used, apply the structured checklist below. Note the relevant service in brackets. Also ask the open-ended question at the end.

38.      Reasons for non-utilization (tick all that apply; note service in brackets):

           ☐ Did not know where to apply / lack of guidance [___________]

           ☐ Application process too complicated [___________]

           ☐ Required documents not available (specify: __________) [___________]

           ☐ Distance / transport problems [___________]

           ☐ Financial cost (travel, fees, informal payments) [___________]

           ☐ Long waiting times [___________]

           ☐ Staff behaviour / attitude problems [___________]

           ☐ Stigma or embarrassment [___________]

           ☐ Physical inaccessibility of office or facility [___________]

           ☐ Did not feel eligible despite having disability [___________]

           ☐ Service quality poor or not useful [___________]

           ☐ COVID-19 / pandemic-related service disruption [___________]

           ☐ Other: _________________________________ [___________]

           Single biggest barrier: _______________________________________________

39.      What other challenges have you faced in accessing disability services?

___________________________________________________________________

___________________________________________________________________

Signature of Interviewer: ________________________   Date: ______________

Signature / Thumb impression of Respondent: ________________________

Note: The Hindi version of this interview schedule is available from the corresponding author upon request.
